# The degree of recovery in swallowing ability in older inpatients with aspiration pneumonia is related to intramuscular adipose tissue of the quadriceps than to muscle mass

**DOI:** 10.1371/journal.pone.0275810

**Published:** 2022-10-10

**Authors:** Naoki Akazawa, Masaki Kishi, Toshikazu Hino, Ryota Tsuji, Kimiyuki Tamura, Akemi Hioka, Hideki Moriyama

**Affiliations:** 1 Department of Physical Therapy, Faculty of Health and Welfare, Tokushima Bunri University, Tokushima, Tokushima, Japan; 2 Department of Rehabilitation, Kasei Tamura Hospital, Wakayama, Wakayama, Japan; 3 Life and Medical Sciences Area, Health Sciences Discipline, Kobe University, Kobe, Hyogo, Japan; Ehime University Graduate School of Medicine, JAPAN

## Abstract

**Background & aim:**

A recent study reported that the increase in intramuscular adipose tissue of the quadriceps in older inpatients is related to a decreasing degree of recovery in swallowing ability compared to the loss of muscle mass. However, whether the association remains true in case of aspiration pneumonia is unclear. Therefore, this study aimed to examine the relationship between the degree of recovery in swallowing ability and intramuscular adipose tissue in the quadriceps of older inpatients with aspiration pneumonia.

**Methods:**

This prospective study included 39 older patients with aspiration pneumonia. Swallowing ability was assessed using the Food Intake Level Scale (FILS). The indicators for the degree of recovery in swallowing ability were FILS at discharge and change in FILS. A greater change in FILS indicates a greater improvement in swallowing ability. Intramuscular adipose tissue and muscle mass of the quadriceps were evaluated at admission using echo intensity and muscle thickness on ultrasound images, respectively. Multiple regression analysis was used to determine whether the echo intensity of the quadriceps was independently and significantly related to FILS at discharge and the change in FILS. Independent variables were age, sex, days from disease onset, echo intensity and muscle thickness of the quadriceps, subcutaneous fat thickness of the thigh, FILS at admission, and number of units of rehabilitation therapy.

**Results:**

Echo intensity of the quadriceps (β = −0.363, p = 0.012) and FILS at admission (β = 0.556, p < 0.001) were independently and significantly associated with FILS at discharge (R^2^ = 0.760, f^2^ = 3.167, statistical power = 1.000). Similar variables (echo intensity of the quadriceps [β = −0.498, p = 0.012] and FILS at admission [β = −0.635, p < 0.001]) were independently and significantly related to change in FILS (R^2^ = 0.547, f^2^ = 1.208, statistical power = 0.998). Quadriceps muscle thickness was not independently and significantly related to FILS at discharge and change in FILS.

**Conclusion:**

Our results indicate that intramuscular adipose tissue of the quadriceps in older inpatients with aspiration pneumonia is more strongly related to the degree of recovery in swallowing ability (that is, swallowing ability at discharge and change in swallowing ability) than muscle mass, and patients who have high intramuscular adipose tissue of the quadriceps at admission have a lower degree of recovery in swallowing ability.

## Introduction

The prevalence of aspiration pneumonia increases with age [1]. In addition, aspiration pneumonia is frequently observed in clinical settings [2, 3] and is related to the mortality of older inpatients [4]. Furthermore, previous studies reported that aspiration pneumonia is associated with dysphagia [5, 6] and sarcopenia [7]. Based on the deep relationship between them [8], which is related to declining activities of daily living (ADL) [9, 10] and increased mortality [11, 12], improving dysphagia and sarcopenia is important for improving the prognosis of older inpatients with aspiration pneumonia.

Recently, swallowing-related muscles as well as appendicular and lower extremity muscles have been shown to be related to swallowing ability [13–15]. Some studies [15, 16] reported that the degree of recovery in swallowing ability in patients with high muscle mass in the appendicular and lower extremities is higher than that for patients with less muscle mass. Furthermore, it has been reported that chair-stand exercises improve swallowing ability [17], and aggressive gait training for patients with nasogastric tube feeding or gastrostomy increases the recovery of oral feeding [18]. Based on these findings, not only swallowing-related muscles but also whole-body muscles, especially the lower extremity muscles, are considered to be important for improving swallowing ability.

The European Working Group on Sarcopenia in Older People proposed the importance of assessing muscle mass as well as muscle quality, including intramuscular adipose tissue, in sarcopenia diagnosis [19]. A recent study [20] reported that intramuscular adipose tissue of the tongue in older persons is related to tongue pressure rather than muscle mass. In addition, because muscle mass of the lower extremity is related to swallowing ability, we examined the relationships between muscle mass and intramuscular adipose tissue of the quadriceps and the degree of recovery of swallowing ability in older inpatients [21]. The results indicated that the increase in intramuscular adipose tissue of the quadriceps in older inpatients was related to the decreasing degree of recovery in swallowing ability compared to the loss of muscle mass [21]. However, whether the intramuscular adipose tissue of the quadriceps in older inpatients with aspiration pneumonia is related to the degree of recovery in swallowing ability remains unclear. Examining this relationship is important for exploring an effective approach to improve the swallowing ability in these patients. This study aimed to examine the relationship between the degree of recovery in swallowing ability and intramuscular adipose tissue in the quadriceps of older inpatients with aspiration pneumonia.

## Materials and methods

### Study design and participants

This prospective study included older patients with aspiration pneumonia who were referred to the Department of Rehabilitation at Kasei Tamura Hospital. Aspiration pneumonia was diagnosed on the basis of symptoms (fever, phlegm, and cough), inflammatory markers, chest imaging (X-ray and computed tomography), and swallowing status. The exclusion criteria of this study were age < 65 years, lack of data, and hospital admission due to the onset of other diseases. Patients with aspiration pneumonia who died during hospital stay were also excluded. A total of 455 inpatients were included in this study. Of these, patients who were < 65 years of age (n = 33) or lacked the necessary data (n = 18) were excluded. We also excluded 363 patients who were admitted to the hospital because of the onset of other diseases (stroke: 60, fracture: 126, heart disease: 25, spinal cord disease: 15, urinary tract infection: 12, and others: 125). In addition, two patients with aspiration pneumonia were excluded because of death during the hospital stay. Ultimately, 39 patients with aspiration pneumonia were included in the study. Rehabilitation therapy, including physical therapy, occupational therapy, and speech and swallowing therapy, was administered to all the participants during hospitalization. All participants or their guardians provided written informed consent prior to the study, which was approved by the ethics committee of Tokushima Bunri University.

### Outcome measures

The main outcome of this study was the degree of recovery in swallowing ability. We also measured the characteristics of the participants at admission, including age, sex, body weight, height, body mass index (BMI), intramuscular adipose tissue and muscle mass of the quadriceps, subcutaneous fat mass of the thigh, nutritional status, inflammation, comorbidities, number of medications, number of units of rehabilitation therapy (1 unit of rehabilitation therapy = 20 min), and ADL. The length of hospital stay (days) and days from disease onset were assessed at discharge. The length of hospital stay was evaluated based on the hospitalization period at Kasei Tamura Hospital. Among the 39 patients, 11 (28.2%) were initially admitted to other hospitals; the days from the onset of disease in these patients were calculated by summing the lengths of both hospital stays.

### Assessment of swallowing ability

Swallowing ability was assessed using the Food Intake Level Scale (FILS). The FILS is a 10-point observer-rated scale [22]. Levels 1 to 3 display various degrees of non-oral feeding; levels 4 to 6 relate to various degrees of oral food intake and alternative nutrition, such as enteral and parenteral nutrition; levels 7 to 9 refer to various degrees of oral intake alone; and level 10 displays normal oral food intake [22]. The validity and reliability of the FILS were reported in a previous study [22]. The indicators for the degree of recovery in swallowing ability were FILS at discharge and change in FILS. The change in FILS was calculated by subtracting FILS at admission from FILS at discharge. A greater change in FILS indicates a greater improvement in swallowing ability.

### Measurements of intramuscular adipose tissue and muscle mass in the quadriceps and subcutaneous fat mass of the thigh

**Fig 1:**
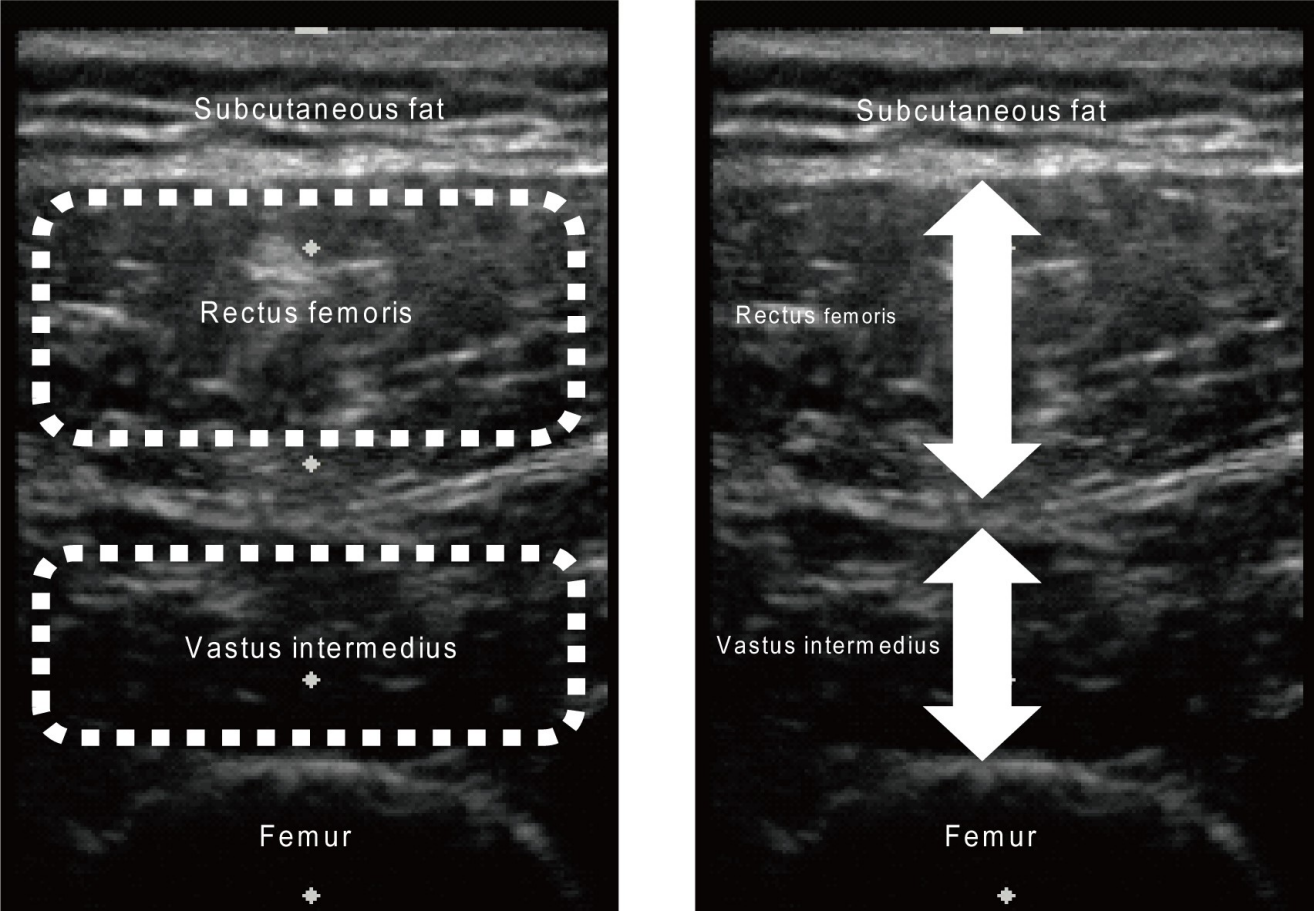
Measurement areas of echo intensity (dashed lines) and muscle thickness (arrows) of the rectus femoris and vastus intermedius.

Transverse ultrasound images were obtained using a B-mode ultrasound system (NanoMaxx; SonoSite Japan, Tokyo, Japan) with a linear array probe (L25n/13–6 MHz; NanoMaxx). The intramuscular adipose tissue and muscle mass of the rectus femoris and vastus intermedius of all participants were evaluated based on echo intensity and muscle thickness [21, 23–33], respectively. The validity of intramuscular adipose tissue and muscle mass measurements using ultrasound has been confirmed in recent studies using magnetic resonance imaging [34–36]. Images of the rectus femoris and vastus intermedius were obtained at 30% of the distance from the anterior superior iliac spine to the proximal end of the patella [21, 23, 26, 30–33]. The participants laid in a supine position with their lower limbs relaxed while a water-soluble transmission gel was applied to the skin surface of the thigh. The probe was pressed lightly against the skin to prevent muscle deformation. All the ultrasound images were captured by the same investigator. Echo intensity was assessed in the regions of interest to include as much muscle as possible while avoiding the bone and surrounding fascia [21, 23, 26, 30–33]. The thickness of the rectus femoris was determined as the distance between the superficial adipose tissue–muscle interface and the deep muscle–muscle interface [21, 23, 26, 30–33], while that of the vastus intermedius was determined as the distance between the superficial muscle–muscle interface and the bone–muscle interface [21, 23, 26, 30–33]. Fig 1 shows the measurement areas of echo intensity and thickness of the rectus femoris and vastus intermedius. Muscle echo intensity and thickness were measured using the ImageJ 1.49 software (National Institutes of Health, Bethesda, MD, USA) [21, 23, 25–28, 30–33]. Echo intensity was examined by performing a computer-assisted 8-bit gray-scale analysis, and the mean echo intensity of the regions of interest was rated from 0 (black) to 255 (white) [21, 23–33]. A higher echo intensity indicates greater intramuscular adipose tissue [37].

The echo intensity of the quadriceps was calculated as the mean echo intensity of the rectus femoris and vastus intermedius. Mean echo intensities of the right and left quadriceps were used in the analysis. The sum of the thicknesses of the rectus femoris and vastus intermedius was used as quadriceps thickness. The mean quadriceps thickness on the right and left sides was included in the analysis. Measurements of the rectus femoris and vastus intermedius echo intensities and muscle thicknesses have relatively high reliability (intraclass correlation coefficients [1.1], 0.857–0.959) [23]. Subcutaneous fat mass in the thigh was assessed based on the subcutaneous fat thickness, which was defined as the distance between the dermis and adipose tissue interface and the muscle–adipose tissue interface [21, 23, 26, 30–33]. The mean subcutaneous fat thickness of the right and left thighs was used in the analysis.

### Measures of other characteristics

Nutritional status was evaluated using the Geriatric Nutritional Risk Index (GNRI) score [38]. The GNRI score was calculated using the following formula: GNRI score = (14.89 × serum albumin [g/dL]) + (41.7 × body weight [kg]/ideal body weight) [38]. Inflammatory status was assessed using the C-reactive protein (CRP) concentration. Comorbidities were assessed using the updated Charlson Comorbidity Index (UCCI) score [39]. ADL was evaluated using the Barthel Index (BI) [40]. BI is widely used in clinical settings and includes ordinal assessments (0–100 points) [40]. Lower BI scores indicate a poor ability to perform ADL.

### Sample size calculation

The effect size (f^2^) of the multiple regression analysis in a recent study [21], which reported that an increase in intramuscular adipose tissue of the quadriceps in older inpatients is related to a decreasing degree of recovery in swallowing ability, was 1.512 (number of dependent variables = 13, R^2^ = 0.602). We speculated that a similar degree of relationship would be observed between intramuscular adipose tissue of the quadriceps and the degree of recovery in the swallowing ability in older patients with aspiration pneumonia. Sample size calculation based on an effect size (f^2^) of 1.512, 15 dependent variables, a statistical power of 0.80, and an alpha error of 0.05, indicated that 28 participants were required. The sample size in this study was calculated using G*Power version 3.1.9.2 (Heinrich-Heine-Universität Düsseldorf, Düsseldorf, Germany).

### Statistical analysis

All statistical analyses were conducted using the SPSS version 28 software (IBM SPSS Japan, Tokyo, Japan). The normality of the variables was assessed using the Shapiro–Wilk test. Parametric data are reported as mean ± standard deviation, whereas nonparametric data are expressed as median (interquartile range [IQR]).

The FILS at admission and discharge were compared using the Wilcoxon signed-rank test. The associations of echo intensity and muscle thickness of the quadriceps with FILS at admission and discharge and change in FILS were determined using Kendall’s τ rank correlation coefficient. Subcutaneous fat thickness affects echo intensity [41]. Therefore, we also examined the associations of echo intensity of the quadriceps with FILS at admission and discharge and change in FILS using partial correlation analysis adjusted for subcutaneous fat thickness of the thigh. Multiple regression analysis (forced entry method) was used to determine whether the echo intensity of the quadriceps was independently and significantly related to FILS at discharge and change in FILS. The independent variables were age, sex (male = 1, female = 2), days from disease onset, echo intensity and muscle thickness of the quadriceps, subcutaneous fat thickness of the thigh, FILS at admission, and number of units of rehabilitation therapy. The variance inflation factor was used to assess multicollinearity; a value > 10 was considered indicative of the presence of multicollinearity. P < 0.05 was considered to express statistical significance. In addition, we calculated the effect size (f^2^) of the multiple regression analysis for FILS at discharge and change in FILS using the following equation: R^2^/ (1 − R^2^). The statistical power of the analysis was calculated using G* Power version 3.1.9.2, based on f^2^, an alpha error of 0.05, the total sample size, and a number of predictor variables.

## Results

The medians (IQR) of FILS at admission, FILS at discharge, and change in FILS were 7.0 (6.0–7.0), 7.0 (6.0–7.0), and 0.0 (0.0–1.0), respectively. There was no significant difference between FILS at admission and discharge (p = 0.310). Improvement, preservation, and deterioration of FILS were observed in 11 (28.2%), 21 (53.8%), and seven (17.9%) patients, respectively. The median (IQR) of days from disease onset and length of hospital stay were 77.0 (49.0–93.0) and 70.0 (37.0–92.0), respectively. Table 1 presents the characteristics of the participants.

**Table 1:** Characteristics of participants at admission.

Characteristics	
Age, years	83.5 ± 7.6
Sex, male/female	26 (66.7)/13 (33.3)
Height, cm	158.0 (149.0–164.0)
Body weight, kg	43.6 (35.2–47.5)
Body mass index, kg/m^2^	17.8 ± 3.2
Quadriceps echo intensity (gray-scale range, 0–255)	92.7 ± 19.7
Quadriceps thickness, cm	1.0 ± 0.4
Subcutaneous fat thickness of the thigh, cm	0.3 ± 0.1
C-reactive protein, mg/dL	1.2 (0.4–6.8)
Serum albumin, g/dL	3.0 ± 0.4
Geriatric Nutritional Risk Index score	77.4 ± 9.0
Updated Charlson comorbidity index score	2.0 (2.0–3.0)
Number of medications	5.0 (3.0–8.0)
Number of rehabilitation therapies, units/day	3.0 (2.0–4.0)
Barthel Index score	15.0 (0.0–35.0)

Data are presented as the mean ± standard deviation, n (%), or median (interquartile range).

**Fig 2:**
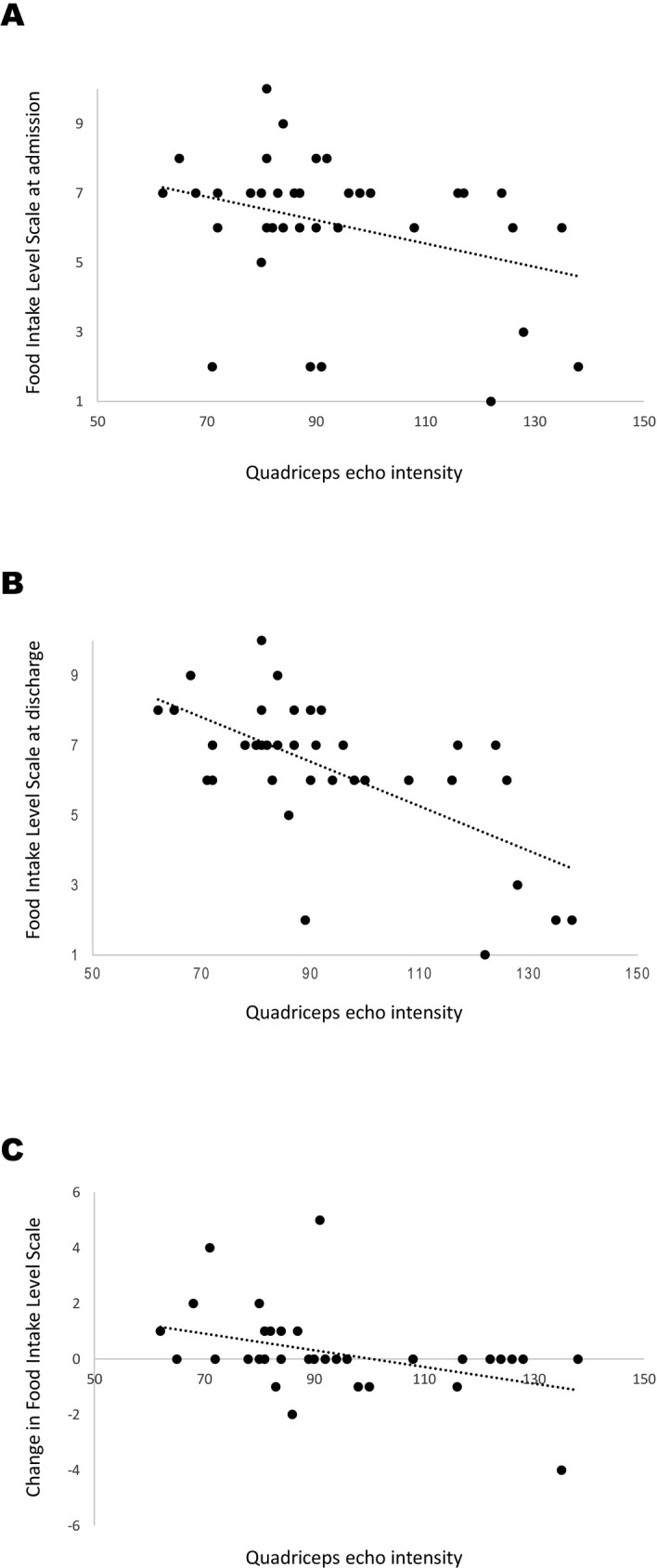
Scatter plots between echo intensity of the quadriceps and Food Intake Level Scale at admission (A) and discharge (B), and change in Food Intake Level Scale (C).

Table 2 presents the results of the correlation analysis. In the correlation analysis, the echo intensity of the quadriceps was significantly related to FILS at discharge and change in FILS, but was not significantly related to FILS at admission. Fig 2 shows the scatter plots between the echo intensity of the quadriceps and FILS at admission and discharge, and the change in FILS. Muscle thickness of the quadriceps was significantly related to FILS at discharge but was not to FILS at admission or change in FILS. In the partial correlation analysis, echo intensity of the quadriceps was significantly related to FILS at admission and discharge and to change in FILS. Tables 3 and 4 present the results of multiple regression analysis. Echo intensity of the quadriceps (β = −0.363, p = 0.012) and FILS at admission (β = 0.556, p < 0.001) were independently and significantly associated with FILS at discharge (R^2^ = 0.760, f^2^ = 3.167, statistical power = 1.000) (Table 3). Similar variables (echo intensity of the quadriceps [β = −0.498, p = 0.012] and FILS at admission [β = −0.635, p < 0.001]) were independently and significantly related to change in FILS (R^2^ = 0.547, f^2^ = 1.208, statistical power = 0.998) (Table 4). Quadriceps muscle thickness was not independently and significantly related to FILS at discharge and to change in FILS.

**Table 2:** Associations of echo intensity and muscle thickness of the quadriceps with Food Intake Level Scale at admission and discharge and with change in Food Intake Level Scale.

Variables	Food Intake Level Scale at admission	p-value	Food Intake Level Scale at discharge	p-value	Change in Food Intake Level Scale	p-value
Quadriceps echo intensity at admission	−0.19^a^	0.132	−0.42^a^	< 0.001	−0.36^a^	0.004
Quadriceps echo intensity at admission	−0.38^b^	0.019	−0.64^b^	< 0.001	−0.37^b^	0.024
Quadriceps thickness at admission	0.22^a^	0.086	0.29^a^	0.021	0.12^a^	0.357

^a^Kendall’s τ rank correlation coefficient.

^b^partial correlation coefficient adjusted for subcutaneous fat thickness of the thigh.

**Table 3:** Multiple regression analysis for Food Intake Level Scale at discharge.

	B	SE	95% confidence interval of B	β	VIF	p-value
Age	−0.02	0.03	−0.07, 0.04	−0.07	1.26	0.519
Sex	0.66	0.43	−0.21, 1.54	0.16	1.34	0.132
Days from onset of disease	−0.01	0.01	−0.02, 0.00	−0.19	1.46	0.092
Quadriceps thickness at admission	0.36	0.81	−1.30, 2.02	0.07	3.39	0.660
Quadriceps echo intensity at admission	−0.04	0.01	−0.06, −0.01	−0.36	2.32	0.012
Subcutaneous fat thickness of the thigh at admission	−0.18	2.28	−4.82, 4.47	−0.01	2.30	0.939
Food Intake Level Scale at admission	0.55	0.10	0.33, 0.76	0.56	1.39	< 0.001
Number of rehabilitation therapy	0.06	0.16	−0.26, 0.38	0.04	1.53	0.700

B, partial regression coefficient; SE, standard error; β, standardized partial regression coefficient; VIF, variance inflation factor.

**Table 4:** Multiple regression analysis for change in Food Intake Level Scale.

	B	SE	95% confidence interval of B	β	VIF	p-value
Age	−0.02	0.03	−0.07, 0.04	−0.09	1.26	0.519
Sex	0.66	0.43	−0.21, 1.54	0.22	1.34	0.132
Days from onset of disease	−0.01	0.01	−0.02, 0.00	−0.26	1.46	0.092
Quadriceps thickness at admission	0.36	0.81	−1.30, 2.02	0.10	3.39	0.660
Quadriceps echo intensity at admission	−0.04	0.01	−0.06, −0.01	−0.50	2.32	0.012
Subcutaneous fat thickness of the thigh at admission	−0.18	2.28	−4.82, 4.47	−0.01	2.30	0.939
Food Intake Level Scale at admission	−0.45	0.10	−0.67, −0.24	−0.64	1.39	< 0.001
Number of rehabilitation therapy	0.06	0.16	−0.26, 0.38	0.06	1.53	0.700

B, partial regression coefficient; SE, standard error; β, standardized partial regression coefficient; VIF, variance inflation factor.

## Discussion

Our results indicate that there is a negative relationship between intramuscular adipose tissue of the quadriceps at admission and the degree of recovery in swallowing ability in older inpatients with aspiration pneumonia. In contrast, there was no relationship between the muscle mass of the quadriceps and the degree of recovery in swallowing ability. Taken together, the intramuscular adipose tissue of the quadriceps in older inpatients with aspiration pneumonia is more strongly related to the degree of recovery in swallowing ability (that is, swallowing ability at discharge and change in swallowing ability) than muscle mass, and patients with high intramuscular adipose tissue of the quadriceps at admission have a lower degree of recovery in swallowing ability.

Based on the results of this study, intramuscular adipose tissue of the quadriceps in older inpatients with aspiration pneumonia is considered a predictor of the degree of recovery in swallowing ability compared to muscle mass. With respect to the background of this result, two points were considered. First, the values might have overestimated the muscle mass because these values include not only actual muscle mass but also the mass of intramuscular adipose tissues [24, 42]. Second, previous studies reported that swallowing ability is related to appendicular skeletal muscle mass [13, 43]. Additionally, appendicular skeletal muscle mass is related to knee extension strength [44, 45], gait ability [46], and ADL [47, 48]. However, intramuscular adipose tissue in the quadriceps shows increased association with knee extension strength [25–27], gait ability [28–30], and ADL [31, 32] as compared to muscle mass. These findings indicate that in contrast to muscle mass, intramuscular adipose tissue of the quadriceps is indirectly related to swallowing ability. These two points were considered the background to our results.

Recently, intervention for lower extremities has been shown to contribute to the recovery of swallowing ability [17, 18]. The degree of recovery in swallowing ability has been reported to be high in stroke patients who perform more chair-stand exercises [17]. Furthermore, aggressive gait training for patients with nasogastric tube feeding or gastrostomy using orthoses that is, knee–ankle–foot orthosis, trunk–hip–bilateral knee–ankle–foot orthoses, and knee orthosis increases the recovery of oral feeding in these patients [18]. Our results, in which intramuscular adipose tissue of the quadriceps is related to the degree of recovery in swallowing ability in older patients with aspiration pneumonia, support these recent findings. Considering our results, intervention for intramuscular adipose tissue in the quadriceps may be needed to improve the swallowing ability of older inpatients with aspiration pneumonia.

Physical activity intervention is reported to be effective in improving intramuscular adipose tissue of the thigh in older frail persons [49]. Considering this, physical activity intervention may be an effective approach for improving intramuscular adipose tissue in the quadriceps in older inpatients with aspiration pneumonia. Ishii et al. [50] targeted older patients with low ADL and reported that the swallowing ability in older patients who spent 4 or more hours away from the bed in a day was higher than that of older patients who spent less than 4 hours away from the bed. The results of this study indicate that there is a relationship between physical activity and swallowing ability, and thus, support our hypothesis (intervention with physical activity for decreasing intramuscular adipose tissue of the quadriceps is needed to improve swallowing ability in older patients with aspiration pneumonia).

A score below 60 in BI is interpreted as a severe dependency condition [51]. The median BI score at admission of the participants in this study was 15. In other words, the decrease in ADL of the participants was remarkable. The mean BMI of the participants was 17.8 kg/m^2^. This value was the same as the cut-off value (17.8 kg/m^2^) for severely low BMI in Asians in the Global Leadership Initiative on Malnutrition criteria [33, 52]. Furthermore, the mean GNRI score of the participants was 77.4. This value was lower than the cut-off value (GNRI score of 82) for major malnutrition risk [38]. Although some studies [53, 54] have reported that resistance training improves intramuscular adipose tissue of the quadriceps, conducting resistance training is difficult for older inpatients with aspiration pneumonia who have low ADL and BMI and major malnutrition risk. Physical activity intervention may be realistic for improving intramuscular adipose tissue in the quadriceps of older inpatients with aspiration pneumonia.

The results of this study showed no statistically significant difference between FILS at admission and discharge, and the FILS of many participants indicated preservation (53.8%) or deterioration (17.9%) during hospitalization. Old age [55, 56], malnutrition risk [57], low weight [56], and low ADL [55] have been known to negatively affect the recovery of swallowing ability. Considering that older patients with aspiration pneumonia in this study had malnutrition risk, low weight, and low ADL, confirming no difference between FILS at admission and discharge might be a valid result. In addition, swallowing ability is closely related to ADL [55, 58–60]. A previous study [61] targeting older patients with aspiration pneumonia who received rehabilitation indicated that the changes in BI score during hospitalization in almost all participants (71.1%) was < 0, and ADL was less likely to improve. Considering the results of the previous study [61] and our study, swallowing ability in older patients with aspiration pneumonia may be difficult to improve, similar to ADL.

The Special Interest Group on Sarcopenia in the International Society of Physical and Rehabilitation Medicine has recommended using quadriceps thickness obtained from ultrasound images to assess muscle mass in sarcopenia diagnosis [62]. In addition, the effects of aging and disuse are remarkably observed in the quadriceps of the upper and lower extremity muscles [63, 64]. Furthermore, intramuscular adipose tissue in the quadriceps of older inpatients has been reported to be related to gait ability [28–30], ADL [31, 32], and the onset of hospital-associated complications [65]. Based on these findings, targeting the quadriceps was considered valid in this study.

This study had three limitations. First, it was a prospective study. Therefore, the causal relationship between swallowing ability and intramuscular adipose tissue in the quadriceps of older inpatients with aspiration pneumonia remains unclear. A randomized controlled trial is needed to reveal this relationship. Second, the sample size of the study was small. However, in the post-hoc analysis, the statistical powers of the multiple regression analysis for FILS at discharge and change in FILS were 1.000 and 0.998, respectively. In other words, there were no type 2 errors in the statistical analyses in this study. Finally, smoking history has been shown to negatively affect the recovery of swallowing function [66]. However, this study did not examine the smoking history of participants. Therefore, we were unable to treat smoking history as a confounding factor in the statistical analysis.

## Conclusions

Our results indicate that the degree of recovery in swallowing ability in older inpatients with aspiration pneumonia is more strongly associated to intramuscular adipose tissue of the quadriceps than to muscle mass, and patients who have higher intramuscular adipose tissue of the quadriceps at admission have a lower degree of recovery in swallowing ability. Intervention for intramuscular adipose tissue of the quadriceps may be needed to improve the swallowing ability of older inpatients with aspiration pneumonia.

## Supporting information

S1 Dataset (XLSX)
